# *In vitro* and *in vivo* mouse follicle development in ovaries and reaggregated ovaries

**DOI:** 10.1530/REP-18-0115

**Published:** 2018-11-13

**Authors:** Belinda K M Lo, Sairah Sheikh, Suzannah A Williams

**Affiliations:** 1 Nuffield Department of Women’s and Reproductive Health, University of Oxford, Women’s Centre, Level 3, John Radcliffe Hospital, Oxford, United Kingdom; 2 IVF Centre, Hong Kong Sanatorium and Hospital, Happy Valley, Hong Kong

## Abstract

Follicle development requires complex and coordinated interactions between both the oocyte and its associated somatic cells. In ovarian dysfunction, follicle development may be abnormal due to defective somatic cell function; for example, premature ovarian insufficiency or malignancies. Replacing defective somatic cells, using the reaggregated ovary (RO) technique, may ‘rescue’ follicle development. ROs containing mature follicles have been generated when transplanted to a host mouse to develop. We have developed a RO culture technique and the aims were to determine how follicle development differed between transplanted and cultured ROs, and the influence of ovarian age (P2 vs P6). Mouse ROs were cultured for 14 days; P2 and P6 ovaries cultured as Controls. Follicle development was compared to ROs transplanted for 14 days and ovaries from P16 and P20 mice. ROs generated from either P2 or P6 exhibited similar follicle development in culture whereas *in vivo* follicle development was more advanced in P6 ROs. Follicles were more developed in cultured ROs than transplanted ROs. However, follicles in cultured ROs and ovaries had smaller oocytes with fewer theca and granulosa cells than *in vivo* counterparts. Our results demonstrate the fluidity of follicle development despite ovary dissociation and that environment is more important to basal lamina formation and theca cell development. Furthermore, follicle development within cultured ROs appears to be independent of oocyte nest breakdown and primordial follicle formation in source ovaries. Our results highlight the need for understanding follicle development *in vitro*, particularly in the development of the RO technique as a potential fertility treatment.

## Introduction

The development of follicles in the ovary requires complex bidirectional interactions between the somatic cells, granulosa cells (GCs) and theca cells (TCs), and the germ cells with certain steps requiring specific endocrine support. Ovarian tissue culture is a valuable tool for observing follicle development over time and is a potential method to develop fertilisable eggs in situations where conventional *in vitro* fertilisation methods are inappropriate (Eppig & O’Brien 1996, O’Brien *et al.* 2003, Telfer *et al.* 2008, Jin *et al.* 2010, Morohaku *et al.* 2016). In situations such as ovarian dysfunction or disease such as malignancy, follicle development within the ovarian tissue may be suboptimal due to defective somatic cell function. However, ovarian germ cells can be isolated and combined with an alternate source of somatic cells to generate a reaggregated ovary (RO), which if transplanted into an immunocompromised host enables the RO to develop follicles over the following 3–4 weeks (Eppig & Wigglesworth 2000, Gittens 2005, Tong *et al.* 2007). ROs generated from E12.5 to P12 ovaries have supported follicle development *in vivo* to the antral follicle stage (Eppig & Wigglesworth 2000, Eppig *et al.* 2002, Gittens 2005, Lei *et al.* 2006, Park *et al.* 2006, Tong *et al.* 2007). Furthermore, offspring have been produced from eggs developed in ROs generated from fetal and neonatal ovaries (Hashimoto *et al.* 1992, Eppig & Wigglesworth 2000). Short-term culture (4 days (4 d) or 10 d) of ROs has also been carried out using either P1 or fetal (E12.5 to E17.5) ovaries respectively (Lei *et al.* 2006, Park *et al.* 2006). However, follicle development between transplanted and cultured neonatal ROs has not been compared.

The definition of a neonate is broad, P0-P7 (Abdi *et al.* 2013), but the ovarian physiology at each age is quite different. For example, at P0-P1, primordial germ cells exist within germ cell nests and the germ cells are in a syncytium connected by cytoplasmic bridges (Pepling & Spradling 2001, Bristol-Gould *et al.* 2006). Prior to birth, these germ cell nests begin to break down into individual oocytes with many oocytes undergoing apoptosis (De Felici *et al.* 1999, Pepling & Spradling 2001, McClellan *et al.* 2003, Pepling *et al.* 2010). However, this process is not complete till P4-P5 (Pepling & Spradling 2001). Older ovaries contain more developed follicles and therefore the resultant RO generated from these ovaries contain more differentiated somatic cells such as GCs and TCs.

Therefore, we aimed first to determine if follicle development in an RO was affected by the age of the neonatal ovaries and to accomplish this, we compared follicle development in ROs generated from P2 and P6 mice. Second, we aimed to determine how follicle development differed between ROs developed* in vivo* and *in vitro* by comparing whole ovaries developed *in vivo* and *in vitro*.

## Methods

### Animals

All animal studies using mice were carried out with approval by the Local Ethical Review Panel (University of Oxford) under licence, in accordance with the UK Animals (Scientific Procedures) Act 1986. Mice ubiquitously expressing the *LacZ* gene (ROSA*LacZ*) (Friedrich & Soriano 1991) were bred in-house on a mainly C57/BL6 background and CD-1 mice were purchased from Envigo UK. Male ROSA*LacZ* mice were mated with CD-1 mice to generate large litters of F1 pups. F1 pups at postnatal day 2 (P2) and 6 (P6) were cervically dislocated and the ovaries isolated to generate ROs. To allow adequate time for experimental planning and to reduce the number of surplus mice, the minimum neonatal age used was P2. Ovaries were also collected from F1 mice at P2, P6, P16 and P20 for histological analysis.

Immunocompromised B6Rag1 mice were used as recipients for transplantation and were generously donated by Professor Fiona Powrie, Nuffield Department of Medicine, University of Oxford.

### Reaggregated ovaries

The generation of ROs was performed based on Eppig & Wigglesworth (2000), with modifications. For each RO, ovaries from four P2 or P6 mice were collected in warmed Dulbecco’s PBS (DPBS, Sigma) supplemented with 1 mg/mL bovine serum albumin (BSA, Factor V Heat Shock Treated; Fisher Scientific). After the removal of bursa and fat using fine forceps (Dumont #5; Fine Science Tools, Heidelberg, Germany) and microscissors (Fine Science Tools), the ovaries were washed twice in DPBS/BSA.

To dissociate the ovaries into a single cell suspension, the pooled ovaries were digested in 0.05% trypsin and 0.53 mM EDTA (Gibco, ThermoFisher Scientific) supplemented with 0.02% DNase-I (Sigma) and incubated at 37°C for 30 min. Digestion was aided by frequent gentle agitation by pipetting the tissue and solution. After dissociation, the cell suspension was transferred to a 15 mL centrifuge tube containing an equal volume of M199/FBS medium: Medium 199 (M199; Gibco, ThermoFisher Scientific) supplemented with 10% fetal bovine serum (FBS; Labtech, Uckfield, UK), 31.3 mM sodium DL-lactate (Sigma) and 10 mg/mL penicillin-streptomycin (P0781; Sigma). After centrifugation for 5 min at 663 × ***g*** to pellet the cells, the supernatant was discarded, and the cells were resuspended in 3 mL M199/FBS.

The germ and somatic cell population were separated using differential cell adhesion. The resuspended cells were transferred to a tissue culture dish and cultured overnight at 37°C in an atmosphere of 5% CO_2_ and 95% air. After overnight culture, somatic cells adhered to the culture dish (somatic cell population). The germ cell population remained unattached and consisted of germ cells (oocytes), non-viable somatic cells and red blood cells. Unattached cells were carefully removed without dislodging the monolayer underneath and added to a new tissue culture treated dish and cultured with M199/FBS. The adherent somatic cell population was rinsed with DPBS/BSA three times to remove unattached cells. The adherent somatic cell population was removed from the tissue cultured-treated dish using 2 mL of trypsin/EDTA for 5 min. The cells were then transferred to a 15 mL centrifuge tube with 2 mL of M199/FBS and centrifuged at 663 × ***g*** for 5 min to create a cell pellet. The supernatant was discarded, and the cells were resuspended in 3 mL M199/FBS, and this cell suspension was then transferred to a new tissue culture dish. Both germ and somatic cell suspensions were cultured for a further 6 h for a second round of differential adhesion. After the 6 h of culture, both germ and somatic cell populations were collected and centrifuged as previously. The supernatant was removed and each pellet was resuspended in 200 µL M199/FBS. To determine cell numbers, a sample of 10 µL was taken from each cell suspension and mixed with 10 µL trypan blue (Sigma) and assessed using a haemocytometer.

Germ and somatic cells were combined, using 1/3 of germ cells and all somatic cells (this ratio is routinely used for neonatal ROs; Eppig & Wigglesworth 2000). Phytohemagglutinin (PHA-P; Sigma) was added to create a final concentration of 35 µg/mL to promote cell cohesion. After centrifugation of the suspension at 10,000 × ***g*** for 1 min, the reaggregated cells, now described as an RO, were cultured overnight in Waymouth MB752/1 media (Sigma) supplemented with 10% FBS. The ROs were transferred with minimal medium onto a polycarbonate Transwell membrane (6.5 mm diameter, 0.4 µm pore; Corning,) in a 24-well tissue culture treated plate (Corning Costar). To prepare the membrane for overnight culture, 250 µL of Waymouth/FBS medium was added above and below the membrane. Once the RO was transferred to the edge of the membrane, excess media was removed. The 24-well plates containing the ROs were cultured at 37°C with an atmosphere of 5% CO_2_ and 95% air. For transplantation, ROs were transplanted beneath the kidney capsule of ovariectomised immunocompromised mice.

### Ovary culture

After overnight culture, the membrane holding the RO were transferred to a new well containing 300 µL ovary culture media. Both ROs and whole ovaries were cultured for up to 14 days in Waymouth-based culture media: Waymouth medium supplemented with 5% FBS, 2.5 IU recombinant FSH (Gonal-F; Merck-Serono, Middlesex, UK) suspended in minimal essential medium, alpha modification (MEM Alpha; Fisher Scientific), 10 µg/mL BSA (Factor V heat shock treated; Fisher); 1% insulin-transferrin-selenium (ITS-G; Life Technologies, UK), 100 IU/mL penicillin and 0.1 mg/mL streptomycin (Sigma) and 25 µg/mL ascorbic acid (Fisher) suspended in MEM Alpha. The culture media was replaced every 2 days, with excess media used to gently move the RO or ovary and prevent adherence to the membrane. This excess media was removed afterwards to leave a thin film of media covering the ROs or ovaries. The cultures were maintained at 37°C in an incubator infused with 5% CO_2_ and 95% air.

### Tissue collection

Reaggregated ovaries and ovaries were either fixed in 25% glutaraldehyde (Sigma) in 0.1 M PBS (pH 7.3, Sigma) supplemented with 1 M MgCl_2_ for 2 h at room temperature or 10% buffered formalin (Sigma) for 6 h and stored in 70% ethanol. All tissues were dehydrated in increasing concentrations of ethanol and xylene and embedded in paraffin wax.

### Assessment of follicle development

To determine the location and number of follicles present in each ovary or RO, paraffin-embedded tissues were serially sectioned (3 µm) and all 5th sections were stained with haematoxylin (Shandon Gill 2 Haematoxylin; Fisher Scientific) and eosin (Sigma).

Images were taken using a Leica DM 2500 microscope (Microscope Services Ltd., Woodstock, UK) and a MicroPublisher 5.0 RTV camera (Qimaging; Microscope Services Ltd.). Healthy follicles (without nuclear vacuoles or pyknotic bodies) containing an oocyte with clear nuclei were counted in every 5th (cultured ROs and ovaries) or 20th section (*in vivo* ROs and ovaries). In ovaries, primordial and transitional follicles were identified based on oocyte morphology with a single layer of flattened pre-granulosa or a mixture of flattened pre-granulosa and cuboidal GCs; hereafter referred to as primordial follicles. In ROs, putative primordial and transitional follicles were identified based on oocyte morphology; hereafter referred to as putative primordial follicles. Follicles containing at least one full complete layer of cuboidal granulosa cells (GCs) were defined as primary follicles, while secondary follicles contained at least two complete layers of GCs. Preantral follicles had at least three complete layers of GCs and no antrum, while antral follicles had at least three complete GC layers and antrum present. GCs number and TC number was determined. To determine the TC number, TCs with a squamous appearance extending from the follicle basal lamina (FBL) of the follicle up to the visibly different stromal cells were counted (Chiti *et al.* 2017).

To assess FBL definition, sections from ovaries and ROs were stained with Periodic acid-Schiff (PAS; Sigma). FBL integrity of primary, secondary and preantral follicles were classified as ≤50% defined if most of the FBL was thin or indistinguishable from the surrounding stroma or >50% defined if the FBL was clear and surrounding the majority of the follicle. Sections were taken from all conditions and follicles were analysed if the nucleus was visible in the oocyte.

The total number of follicles at each follicle stage in cultured and *in vivo* ovaries and ROs was corrected using the Abercrombie correction factor (Abercrombie 1946).

### Statistical analysis

Analysis was performed using GraphPad Prism version 7.00 (GraphPad Software). Data were tested for normality using D’Agostino & Pearson normality test. Analysis of cell numbers and total follicle numbers in P2 and P6 ovaries prior to culture or manipulation was either performed using t-tests with Welch’s correction (for normally distributed data) or Mann–Whitney U tests (for non-normally distributed data). The ordinary one-way ANOVA with Tukey’s multiple comparisons test was performed to compare primordial follicle numbers between P2, P6, P16 and P20 ovaries. Two-way ANOVAs with Sidak’s multiple comparisons test were performed to compare differences in follicle numbers, at each follicle stage, between different age groups and conditions. Fisher’s exact test was used to analyse the relationship between the FBL definition and the different ovary and RO conditions. The Kruskal–Wallis test with Dunn’s multiple comparisons test was used to compare two variables: the total number of follicles, TC number, GC number, follicle area or oocyte area and different age and/or conditions, at each follicle stage. Results are presented as mean ± s.d., with *P* ≤ 0.05 considered significant.

Linear regressions were performed to correlate follicle area and granulosa area to GC number. Lines shown have *r* ^2^ > 0.6, which indicates the linear model fits >60% of the data plotted.

## Results

### Follicle development in neonatal ovaries

**Figure 1:**
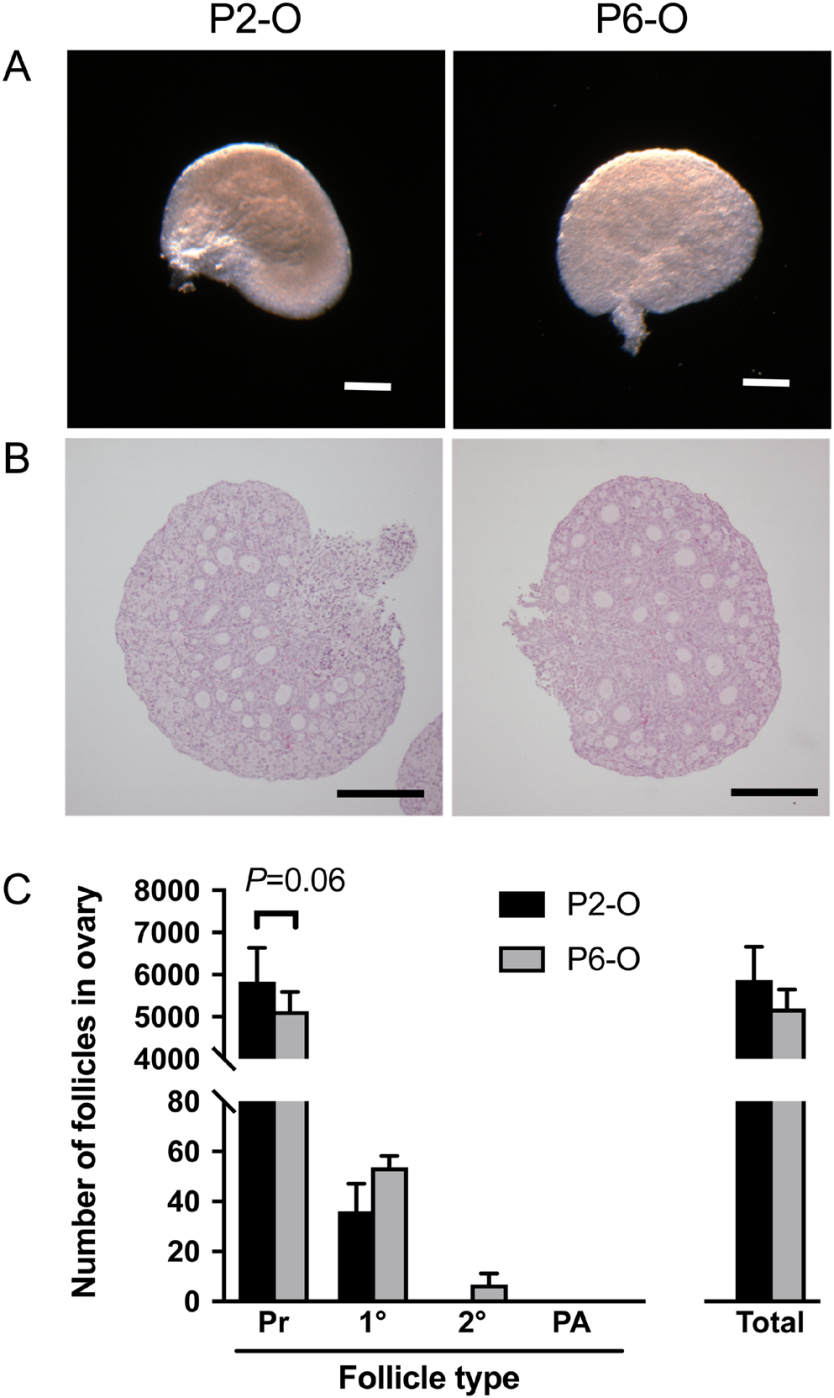
Follicle development in neonatal ovaries. Representative images of (A) neonatal ovaries and (B) central sections stained with haematoxylin and eosin. (C) The number of follicles at each stage of development and the total number of follicles were assessed in neonatal P2 and P6 ovaries. Pr, primordial follicles; 1°, primary follicles; 2°, secondary follicles; PA, preantral follicles. Scale bar: 200 µm. Data are plotted as mean ± s.d. P2-O *n* = 4; P6-O *n* = 3.

It was important to determine the number and stage of the follicles present within P2 and P6 ovaries prior to culture or manipulation (P2-O and P6-O, respectively) (Fig. 1A and B). P2-O contained follicles only up to the primary stage, whereas P6-O contained both primary follicles and secondary follicles (Fig. 1C). Neither P2-O nor P6-O contained preantral follicles.

### Number of germ and somatic cells from digested neonatal ovaries

ROs were generated using ovaries from four neonatal mice (P2 or P6). The ovaries were grouped and digested into a single population and separated into germ and somatic cells. The number of cells was assessed using trypan blue to determine if they were dead or alive; if the cells were stained blue, they were classified as dead. The number of trypan blue-positive dead cells in the germ cell suspension was equivalent between P2 and P6 (P2: 2682 ± 5973; P6: 2171 ± 5520; not significant). The total number of live cells in the germ cell suspension isolated from four mice did not differ between P2 and P6 (P2: 29506 ± 8447; P6: 36914 ± 11490; *P* = 0.06). No dead somatic cells were observed in either age group. As expected, more somatic cells were recovered from the P6 ovaries of four mice compared to the younger P2 ovaries (mean ± s.d.; P2: 342447 ± 124431; P6: 714400 ± 238850; *P* < 0.0001).

### Age-related differences in follicle development in transplanted ROs

Since P6-O contained follicles that were more developed than P2-O, we determined whether transplanted ROs (ROt) generated from P6 ovaries contained follicles at later stages of development compared to ROs generated from P2 ovaries.

**Figure 2:**
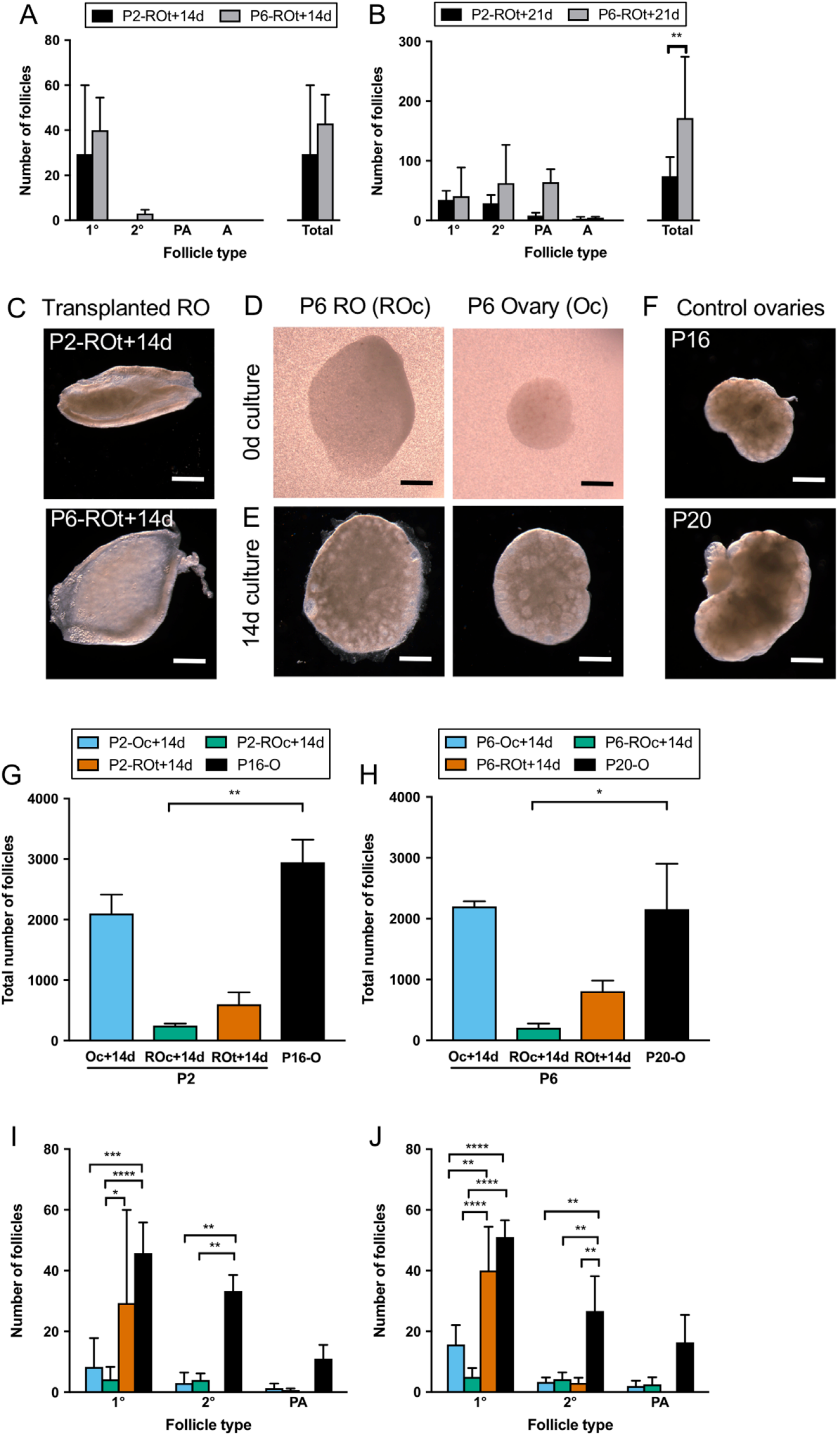
Follicle development in cultured ovaries, cultured reaggregated ovaries, transplanted reaggregated ovaries and age-matched *in vivo* ovaries. Follicle development (follicle stages and total number of developing follicles) in P2 or P6 reaggregated ovaries (ROs) which have been transplanted for (A) 14 days (P2-ROt + 14d and P6-ROt + 14d) and (B) 21 days (P2-ROt + 21d and P6-ROt + 21d) was assessed. Representative images of (C) ROs generated from P2 and P6 ovaries after 14 days of transplantation, (D) ROs and ovaries prior to culture and (E) after 14 days of culture and (F)* in vivo* Control ovaries. (G) Total number of follicles in P2 ovaries (P2-Oc + 14d) and P2 ROs (P2-ROc + 14d) cultured for 14 days, P2 ROs transplanted for 14 days (P2-ROt + 14d) and the age-matched *in vivo* control of P16 ovaries (P16-O) and (H) their equivalent in P6 ovaries and ROs (P6 + Oc + 14d, P6-ROc + 14d, P6-ROt + 14d, P20-O). The total number of follicles for the cultured and transplanted ROs includes putative primordial follicles. (I and J) Follicle development was assessed within these matched P2 and P6 groups. 1°, primary follicles; 2°, secondary follicles; PA, preantral follicles. Scale bar: 500 µm. Data are plotted as mean values ± s.d. P2-Oc + 14d *n* = 3; P2-ROc + 14d *n* = 5; P2-ROt + 14d *n* = 3; P2-ROt + 21d *n* = 3; P16-O *n* = 4; P6-Oc + 14d *n* = 3; P6-ROc + 14d *n* = 4; P6-ROt + 14d *n* = 3; P6-ROt + 21d *n* = 3; P20-O *n* = 3. *P ≤ 0.05; **P ≤ 0.01; ***P ≤ 0.001; ****P ≤ 0.0001.

Analysis of P2 and P6 ROs transplanted for 14 days (P2-ROt + 14d and P6-ROt + 14d) (Fig. 2A) revealed that P2-ROt + 14d contained follicles only up to the primary follicle stage, while P6-ROt +14d contained follicles that were more developed at the secondary follicle stage. Both P2-ROt + 14d and P6-ROt + 14d did not contain preantral or antral follicles and had similar numbers of developing follicles.

Both P2 and P6 ROs transplanted for 21 days (P2-ROt + 21d and P6-ROt + 21d) had follicles at all stages of development up to the antral stage with an increase in the total number of developing follicles (primary follicles onwards) (*P* ≤ 0.01) (Fig. 2B). The use of older ovaries, which contain more mature follicles, in RO generation led to the development of more follicles after 21 days of transplantation.

### Effect of culture on ovarian follicle development and RO follicle development

Since age-modified follicle development in ROs generated *in vivo* (Fig. 2C), we wanted to determine if this modification was also observed in cultured P2 and P6 ROs (ROc: cultured ROs). P2 and P6 ovaries were also cultured without manipulation for 14 days as a control (Oc: cultured ovaries) (Fig. 2D and E). Therefore, follicle development in cultured ROs was compared to ROs generated *in vivo*, and ovaries developed *in vitro* were compared to ovaries developed *in vivo* (Fig. 2F).

The number of developing follicles in both *in vivo* and *in vitro* ROs as well as the cultured ovaries was less than half of those in the *in vivo* developed ovaries for both P2 and P6 (Fig. 2G and H) with total follicle numbers similar between the P2 and P6 groups.

Analysis of the different follicle stages revealed that cultured P2 and P6 ROs and ovaries contained fewer primary and secondary follicles than *in vivo* ovaries (Fig. 2I and J). After 14 days of development, *in vivo* ROs did not contain preantral follicles, whereas cultured ovaries and cultured ROs and *in vivo* ovaries from both age groups did contain follicles at the preantral stage. Despite this difference in transplanted ROs, comparisons between cultured P2 and P6 groups (i.e. P2-Oc + 14d vs P6-Oc + 14d; P2-ROc + 14d vs P6-ROc + 14d) and their respective *in vivo* ovaries revealed no differences in follicle number at each stage of follicle development.

**Figure 3:**
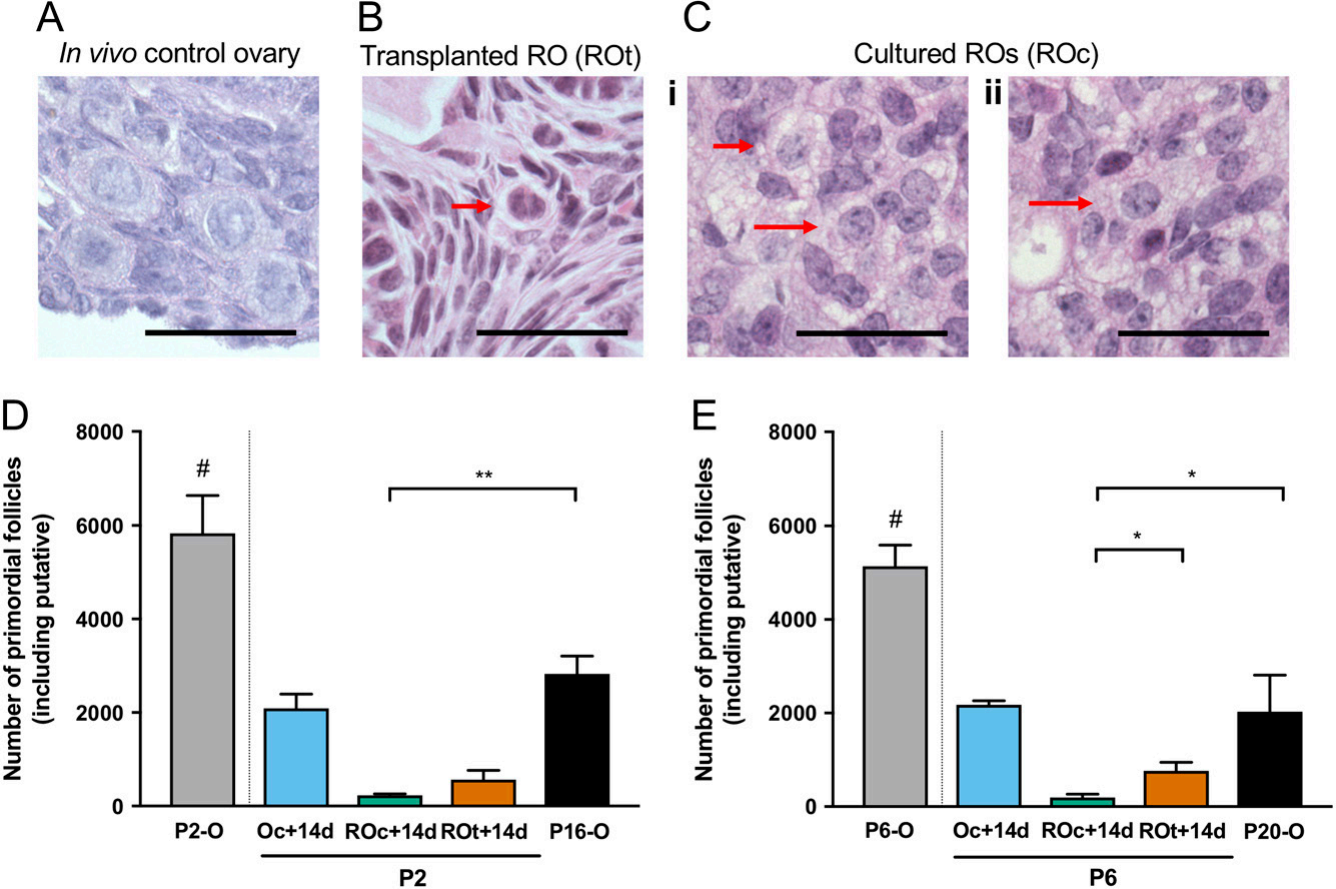
Putative primordial follicles in transplanted and cultured reaggregated ovaries and primordial follicles in age-matched *in vivo* ovaries. Representative images of primordial follicles in (A) P16/P20 *in vivo* Control ovaries and putative primordial follicles in (B) reaggregated ovaries transplanted for 14 days (ROt) and (C) reaggregated ovaries cultured for 14 days (ROc) (red arrows indicate putative primordial follicles). (D) Number of primordial follicles in untreated P2 ovaries (P2-O), P2 ovaries cultured for 14 days (P2-Oc + 14d), age-matched *in vivo* Control of P16 ovaries (P16-O) and putative primordial follicles in P2 ROs (P2-ROc + 14d) cultured for 14 days, P2 ROs transplanted for 14 days (P2-ROt + 14d) and (E) their equivalent in P6 ovaries and ROs (P6-O, P6 + Oc + 14d, P6-ROc + 14d, P6-ROt + 14d, P20-O). ^#^P2-O and P6-O are not compared to the other conditions, as they have not been cultured for 14 days. Data are mean values ± s.d. Scale bar: 50 µm. P2-Oc + 14 days *n* = 3; P2-ROc + 14d *n* = 5; P2-ROt + 14d *n* = 3; P2-ROt + 21d *n* = 3; P16-O *n* = 4; P6-Oc + 14d *n* = 3; P6-ROc + 14d *n* = 4; P6-ROt + 14d *n* = 3; P6-ROt + 21d *n* = 3; P20-O *n* = 3. *P ≤ 0.05; **P ≤ 0.01.

Primordial follicles were readily identified in Control ovaries based on their location towards the cortical region of the ovary and morphology; i.e. oocyte size and the presence of a single layer of flattened pre-GCs (Fig. 3A). Putative primordial follicles in the ROs (Fig. 3B and C) were observed but as expected, were distributed throughout the reaggregated tissue. The putative primordial follicles were identified based on oocyte size and morphology as they lacked the characteristic layer of flattened pre-GCs. As expected, the number of primordial follicles in the ovaries declined with age from P2 to P20 (*P* < 0.0001) with a significant drop between P6 and P16 (*P* < 0.005; Fig. 3D and E). Reduced primordial follicle numbers were observed at both ages in cultured ROs compared to their *in vivo* control ovaries. Additionally, fewer primordial follicles were observed in P6 cultured ROs compared to the P6 transplanted ROs (Fig. 3D and E).

**Figure 4:**
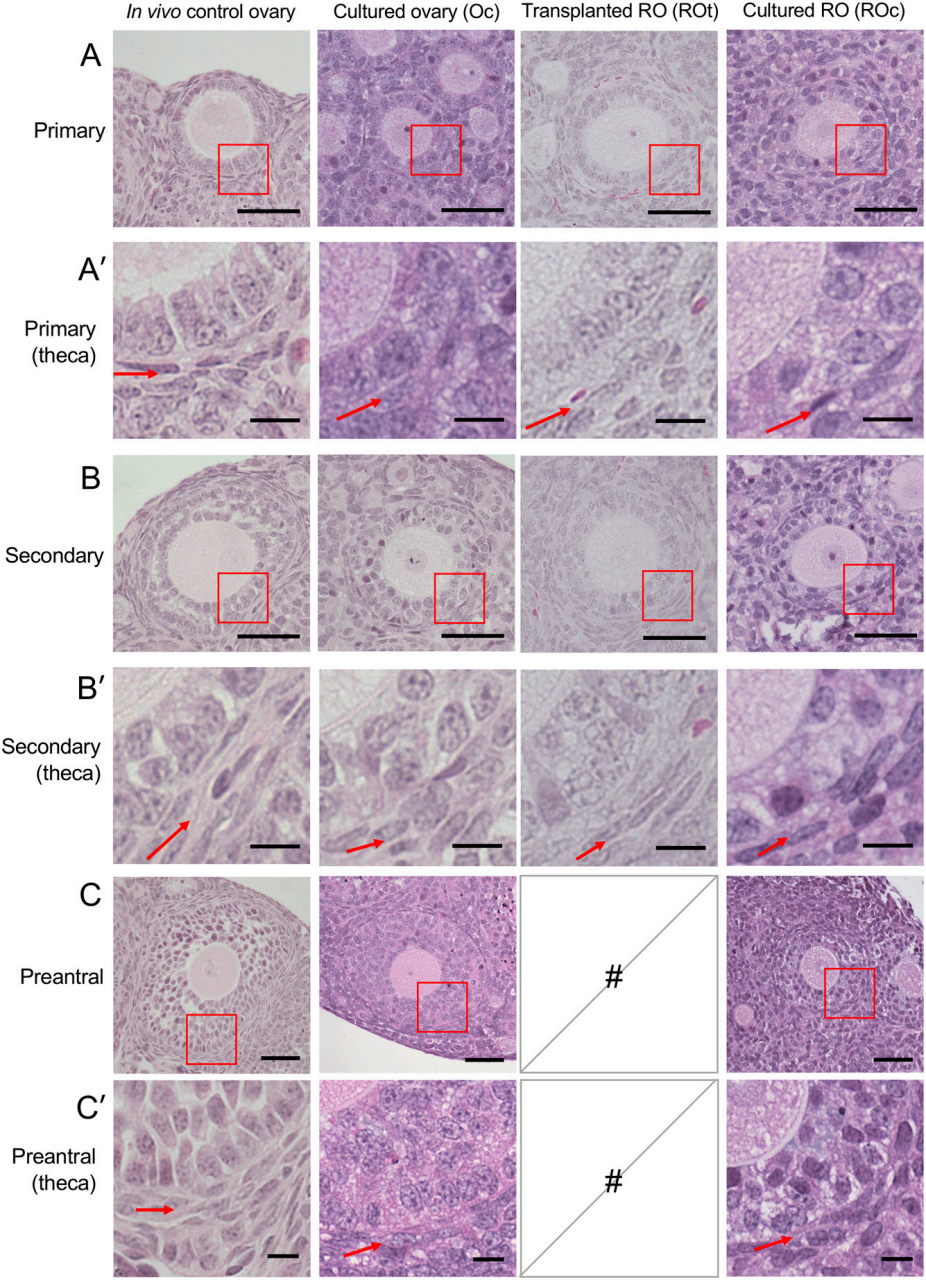
Representative images of follicles at primary, secondary and preantral stages within cultured ovaries, cultured reaggregated ovaries, transplanted reaggregated ovaries and age-matched *in vivo* ovaries. Representative images of a (A) primary follicle from age-matched *in vivo* ovaries, cultured ovaries, transplanted reaggregated ovaries (ROs) and cultured ROs. Red box is magnified in A′. (A′) Higher magnification of the red box in A, showing the theca layer of primary follicles. Red arrows are pointing at the theca layer. Representative images of (B) secondary follicles, (B′) higher magnification of secondary follicle theca layers, (C) preantral follicles and (C′) higher magnification of preantral follicle theca layers are shown. Follicle scale bar (A, B and C): 50 µm; high magnification theca scale bar (A′, B′ and C′): 10 µm. #, no follicles present.

Histological assessment of follicles revealed differences between ovaries and ROs developed *in vivo* and those in the cultured ovaries and ROs (Fig. 2). Primary follicles were analysed (Fig. 4A), and the TC layer appeared to contain fewer TCs in cultured follicles compared to those in *in vivo* ROs and *in vivo* control ovaries (Fig. 4A′). Furthermore, some of the cultured follicles did not appear to have a complete follicle basal lamina (FBL). A similar pattern of fewer TCs and incomplete FBL was observed when secondary (Fig. 4B and B′) and preantral follicles (Fig. 4C and C′) were examined.

**Figure 5:**
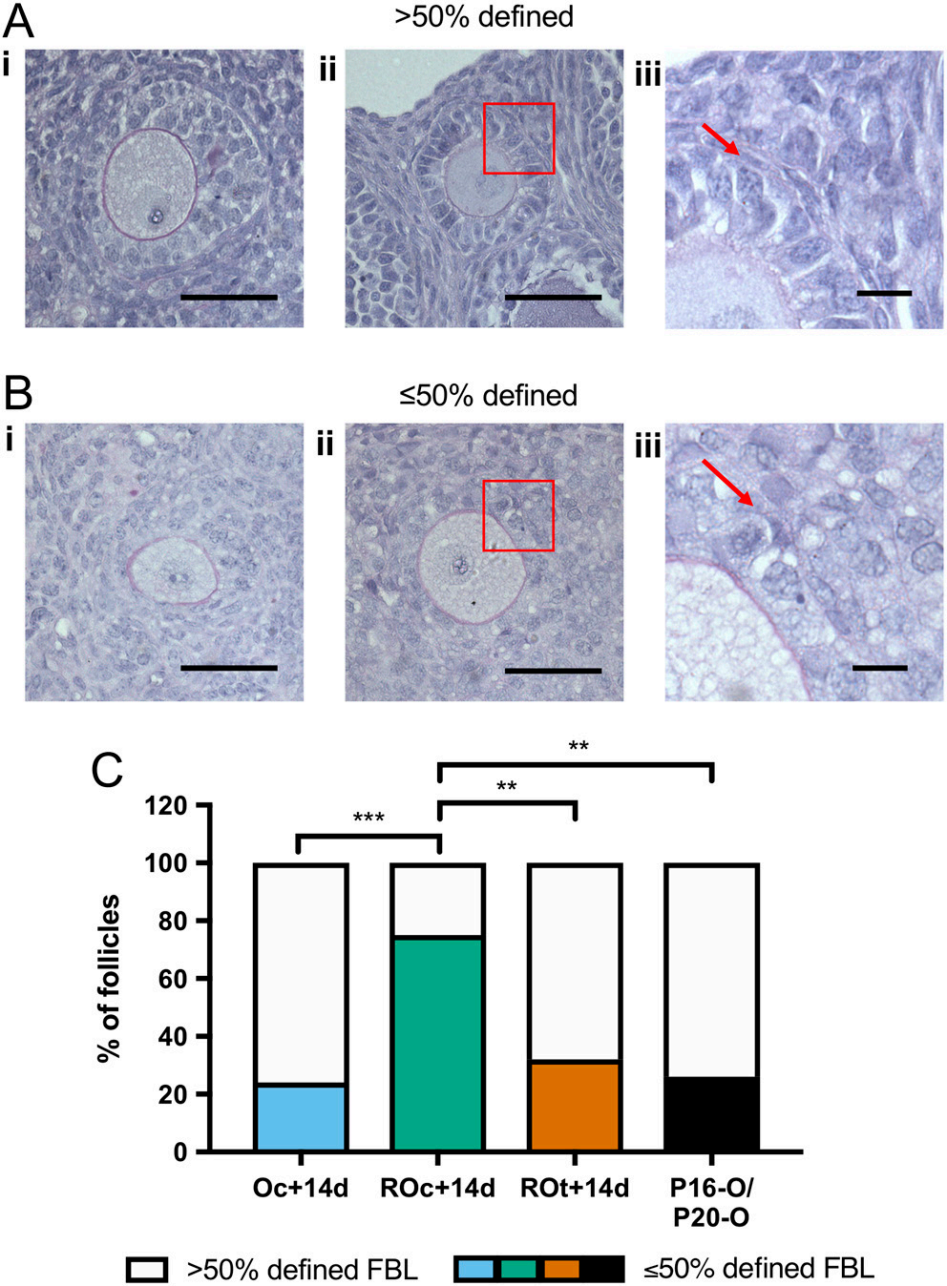
Analysis of basal lamina in ovaries and reaggregated ovaries. (Ai and ii) Representative images of follicles stained with Periodic acid-Schiff and haematoxylin defining how follicle basal lamina (FBL) was classified as >50% defined. (Aiii) Higher magnification of the red box in Aii, with the red arrow pointing at the stained FBL. (Bi, ii) Representative images of follicles classified as ≤50% defined FBL. (Biii) Higher magnification of the red box in Bii, with the red arrow pointing at the lack of FBL definition. (C) Analysis of FBL definition in primary, secondary and preantral follicles in cultured ovaries (Oc + 14d), cultured reaggregated ovaries (ROc + 14d) and transplanted reaggregated ovaries (ROt + 14d) and *in vivo* Control ovaries (P16-O/P20-O). Scale bar (Ai, Aii, Bi and Bii): 50 µm; high magnification of FBL scale bar (Aiii and Biii): 10 µm. P16-O/P20-O *n* = 23 follicles, *n* = 4 ovaries; Oc + 14d *n* = 25 follicles, *n* = 4 ovaries; ROc + 14d *n* = 24 follicles, *n* = 3 ovaries; ROt + 14d *n* = 19 follicles, *n* = 4 ovaries. **P ≤ 0.01; ***P ≤ 0.001.

The integrity of the FBL was assessed in both P2 and P6 conditions. The FBL was classified as >50% defined (Fig. 5Ai and Aii), if the FBL was clearly surrounding most of the follicle (Fig. 5Aiii); or ≤50% (Fig. 5Bi and Bii) if most of the FBL was either thin or indistinguishable from the surrounding stroma (Fig. 5Biii). In P16-O and P20-O, around 20–30% of all follicles were ≤50% FBL defined. This proportion of follicles with ≤50% FBL defined was also observed in the cultured ovary (Oc + 14d) and the transplanted ROs (ROt + 14d). However, the proportion of follicles with a poorly defined FBL in the cultured RO (ROc + 14d) was significantly higher with almost 80% of follicles with ≤50% of the FBL defined.

### Cultured ovaries and ROs are associated with fewer theca and GCs

**Figure 6:**
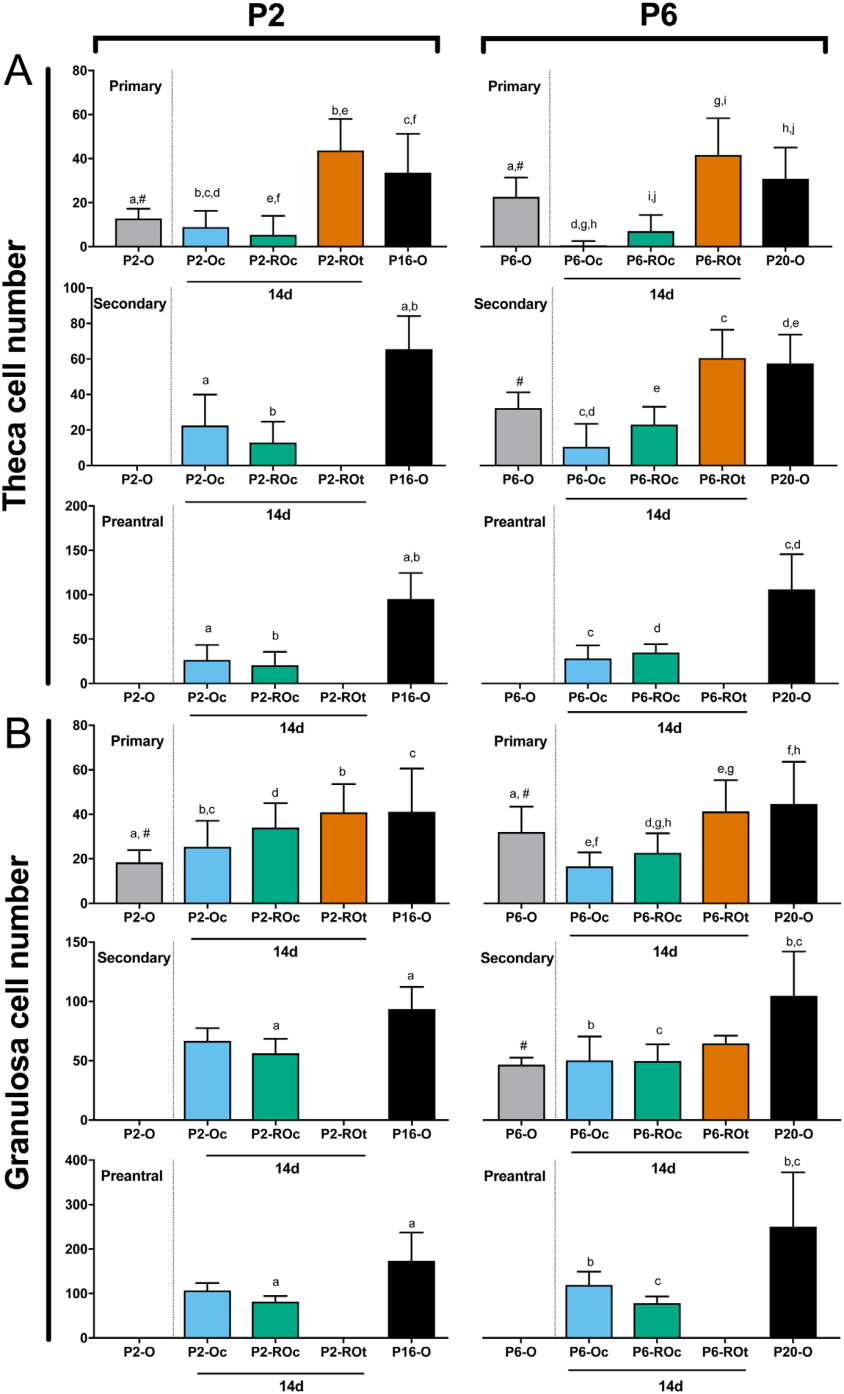
Theca and granulosa cell quantification in ovaries and reaggregated ovaries. Quantification of (A) theca cell number was determined for primary, secondary and preantral follicles in P2 ovaries (P2-O), P2 cultured ovaries (P2-Oc + 14d), P2 cultured reaggregated ovaries (P2-ROc + 14d), P2 transplanted ROs (P2-ROt + 14d) and P16 ovaries (P16-O), as well as for the P6 counterparts (P6-O, P6-Oc + 14d, P6-ROc + 14d, P6-ROt + 14d, P20-O). (B) Granulosa cell number was also determined in primary, secondary and preantral follicles. Data are plotted as mean values ± s.d. Bars with matched lower-case letters are significantly different from each other, within a follicle stage. ^#^P2-O and P6-O are not compared to the other conditions, as they have not been cultured for 14 days. Primary follicles: P2-O *n* = 49; P2-Oc + 14d *n* = 28; P2-ROc + 14d *n* = 109; P2-ROt + 14d *n* = 31; P16-O *n* = 62; P6-O *n* = 57; P6-Oc + 14d *n* = 57; P6-ROc + 14d *n* = 25; P6-ROt + 14d *n* = 44; P20-O *n* = 52. Secondary follicles: P2-Oc + 14d *n* = 12; P2-ROc + 14d *n* = 81; P16-O *n* = 54; P6-O *n* = 9; P6-Oc + 14d *n* = 14; P6-ROc + 14d *n* = 23; P6-ROt + 14d *n* = 4; P20-O *n* = 31. Preantral follicles: P2-Oc + 14d *n* = 5; P2-ROc + 14d *n* = 8; P16-O *n* = 19; P6-Oc + 14d *n* = 11; P6-ROc + 14d *n* = 13; P20-O *n* = 19.

Quantification of TC numbers revealed that follicles from cultured ovaries and cultured ROs had fewer TCs compared to *in vivo* ovaries and *in vivo* developed ROs (Fig. 6A). Although TC numbers in primary follicles in P2-O and P6-O were not compared to the experimental ROs and ovaries, it is interesting to note that primary follicles in P6-O contain more TCs than primary follicles in P2-O. However, this age-related difference did not persist as TC development in cultured or *in vivo* developed P2 and P6 ROs was equivalent.

While the pattern of fewer GCs in cultured tissues was not as pronounced as TCs, primary follicles in cultured ovaries had fewer GCs than follicles from transplanted ROs and in* vivo* ovaries (Fig. 6B). Along with fewer GCs in cultured ovaries, primary follicles in cultured P6 ROs also had fewer GCs compared to their *in vivo* counterparts.

Ovarian age also affected GC number. When comparing P2 and P6 ovaries, the number of GCs in primary follicles was also different with more GCs in P6-O than P2-O (Fig. 6B), consistent with the difference observed in primary follicle TCs (Fig. 6A). It is possible that in this aspect the primary follicles in the P2 ovaries may not be as mature as those in the P6. Unlike TCs, this increase in primary follicle GC numbers was also observed when comparing cultured P6 ROs to cultured P2 ROs.

### Oocyte and follicle area in ovaries and ROs

Since follicles from cultured ovaries and ROs develop fewer TCs and GCs than their *in vivo* counterparts, these follicles may also be developmentally different, despite having the same number of GC layers. Additionally, the germ cells isolated from P6 ovaries are developmentally more advanced than those in P2, as evidenced by the presence of secondary follicles and more primary follicles in P6 ovaries (Fig. 1C). As oocyte diameter has previously been used as a marker for oocyte maturity or meiotic maturity (Griffin *et al.* 2006), comparing oocyte area between *in vitro* and *in vivo* ovaries and ROs may crudely indicate whether the follicles are developmentally comparable.

**Figure 7:**
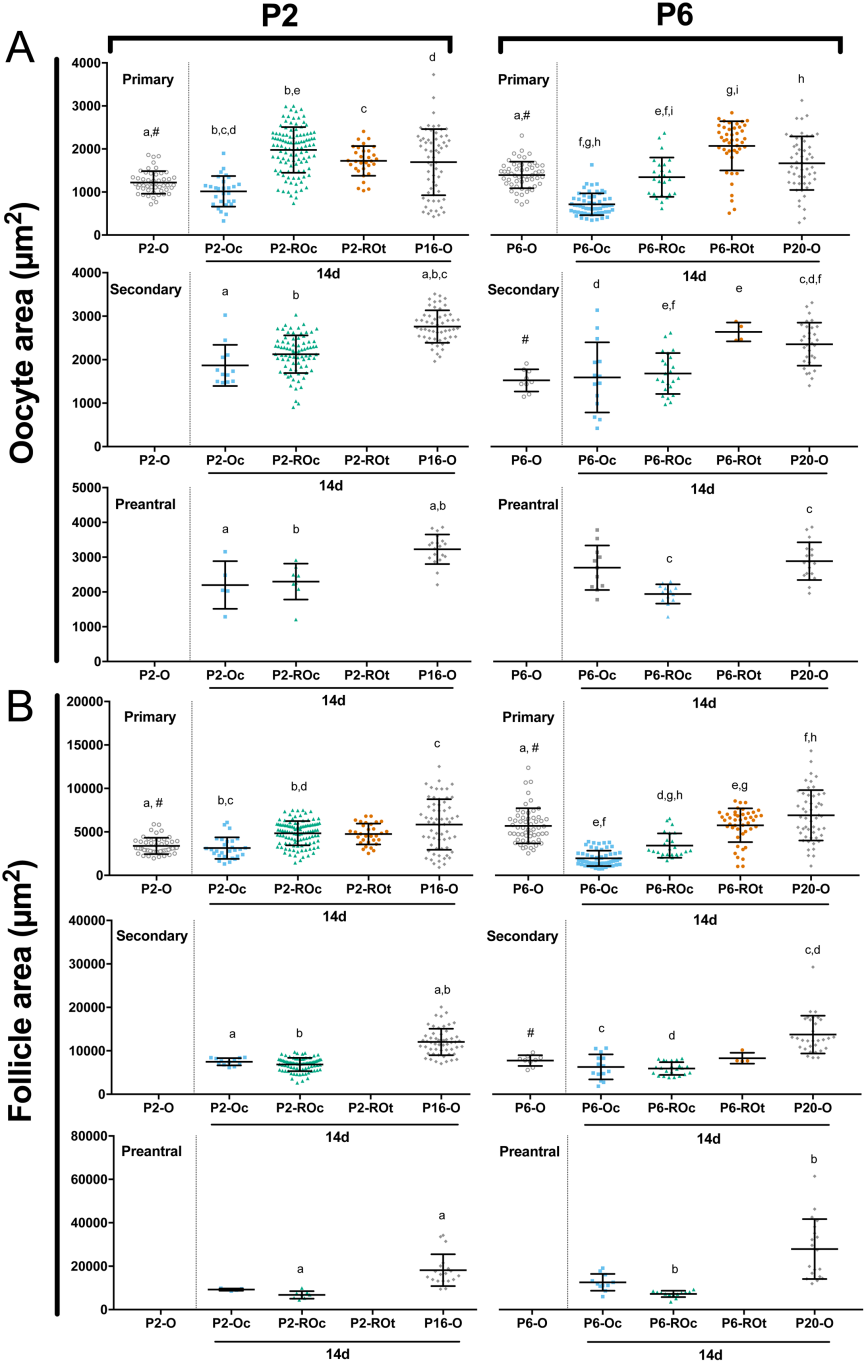
Oocyte and follicle area in ovaries and reaggregated ovaries. (A) Oocyte area and (B) follicle area were determined for primary, secondary and preantral follicles in P2 ovaries (P2-O), P2 cultured ovaries (P2-Oc + 14d), P2 cultured reaggregated ovaries (P2-ROc + 14d), P2 transplanted ROs (P2-ROt + 14d) and P16 ovaries (P16-O), as well as the P6 counterparts (P6-O, P6-Oc + 14d, P6-ROc+14d, P6-ROt + 14 days, P20-O). Data are plotted as mean values ± s.d. Bars with matched lower-case letters are significantly different from each other, within a follicle stage. ^#^P2-O and P6-O are not compared to the other conditions, as they have not been cultured for 14 days. Primary follicles: P2-O *n* = 49; P2-Oc + 14d *n* = 28; P2-ROc + 14d *n* = 109; P2-ROt + 14d *n* = 31; P16-O *n* = 62; P6-O *n* = 57; P6-Oc + 14d *n* = 57; P6-ROc + 14d *n* = 25; P6-ROt + 14d *n* = 44; P20-O *n* = 52. Secondary follicles: P2-Oc + 14d *n* = 12; P2-ROc + 14d *n* = 81; P16-O *n* = 54; P6-O *n* = 9; P6-Oc + 14d *n* = 14; P6-ROc + 14d *n* = 23; P6-ROt + 14d *n* = 4; P20-O *n* = 31. Preantral follicles: P2-Oc + 14d *n* = 5; P2-ROc + 14d *n* = 8; P16-O *n* = 19; P6-Oc + 14d *n* = 11; P6-ROc + 14d *n* = 13; P20-O *n* = 19.

At the primary follicle stage, age affected oocyte size with oocytes from P6-O primary follicles larger than those from P2-O (Fig. 7A). However, when comparing cultured ovaries from P2 and P6 to those developed *in vivo*, all stages of follicle development contained smaller oocytes. Oocytes of primary follicles in cultured ROs were of equivalent size to those developed in transplanted ROs or *in vivo* control ovaries at P2 but not at P6, indicating some effect of tissue age when generated.

Considering the oocyte and GCs contribute to the overall follicle area, any changes in either of the two compartments would affect follicle size at each stage. Since oocyte and GC number were modified in primary follicles, not surprisingly, overall size differed between P2 and P6 ovaries (Fig. 7B). The culture of whole ovaries also resulted in changes to follicle size when compared to *in vivo* developed counterparts with primary and secondary follicles being smaller in the P2 and P6 cultured ovaries. When analysing follicle development in the ROs, there was an effect of age when the ROs were generated. Primary follicle size in cultured P2 ROs did not differ to those grown in transplanted ROs, but at P6, those grown *in vitro* were significantly smaller. Interestingly, age also affected follicle growth in ROs. Primary follicles in cultured P2 ROs were larger than those in cultured ovaries but equivalent in size to transplanted ROs and *in vivo* ovaries, whereas primary follicle size in ROs generated from P6 was reduced in culture compared to those grown in transplanted ROs.

### Correlations between follicle area, granulosa area and GCs

**Figure 8:**
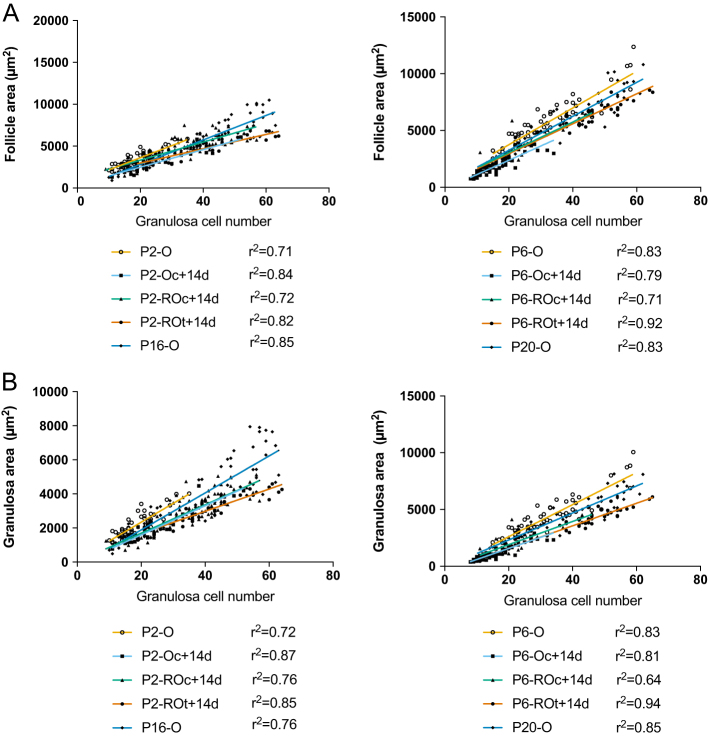
Correlations between follicle and granulosa area to granulosa cell numbers in primary follicles from ovaries and reaggregated ovaries. Linear regressions were performed between (A) follicle area and granulosa cell (GC) number and (B) granulosa area and GC number in primary follicles from P2 whole ovaries (P2-O), P2 cultured ovaries (P2-Oc + 14d), P2 cultured reaggregated ovaries (P2-ROc + 14d), P2 transplanted ROs (P2-ROt + 14d) and P16 ovaries (P16-O), as well as the P6 equivalents (P6-O, P6-Oc + 14d, P6-ROc + 14d, P6-ROt + 14d, P20-O). Primary follicles: P2-O *n* = 49; P2-Oc + 14d *n* = 28; P2-ROc + 14d *n* = 109; P2-ROt + 14d *n* = 31; P16-O *n* = 62; P6-O *n* = 57; P6-Oc + 14d *n* = 57; P6-ROc + 14d *n* = 25; P6-ROt + 14d *n* = 44; P20-O *n* = 52. *r^2^* values are shown next to each line; all graphed lines have *r* ^2^ > 0.6. The lines graphed in follicle area to GC number (P2 group) and granulosa area to GC number (both P2 and P6 group) have significantly different gradients. The lines graphed in follicle area to GC number (P6 group) have similar gradients but differing *y* intercepts.

To determine whether the growth rates of follicles in cultured ROs and ovaries were accelerated compared to transplanted ROs and *in vivo* ovaries, follicle area and granulosa area were assessed relative to GC number. As the number of GCs increases, it is expected that GC area, and thus follicle area, will increase. The rate of area increase per GC can also reflect follicle growth rate. To assess growth using linear regression, primary follicles with a GC number ranging between 9 and 65 (the maximum GC number in transplanted ROs) were analysed. Unsurprisingly, follicle area increased rapidly per GC in all groups from both P2 and P6 (Fig. 6A). However, for P2, the cultured ovaries, *in vitro* and transplanted ROs had slower growth trajectories compared to ovaries developed *in vivo*. This difference in growth trajectories was not evident in the P6 groups (Fig. 8A). Although no difference was observed in growth trajectories of follicle area in the P6 group, both the P2 and P6 groups had different growth trajectories when GC area was assessed (Fig. 8B). Interestingly, both follicle area and GC area per GC increased faster in primary follicles from transplanted P6 ROs compared to P2 ROs (*P* < 0.001; *P* < 0.001). This age-related difference in growth, however, was not observed in the other experimental conditions.

## Discussion

The ability of follicles to reform and develop once the ovary has been dissociated is a powerful technique to study follicle function and also has the potential to be useful in fertility preservation. Developing *in vitro* techniques enables us to explore the dynamics of follicle development, but it is important to determine how follicle development itself is affected by culture. In this study, we investigated follicle development in different ages of neonatal ovaries, how development is affected by culture vs *in vivo*, and finally compared RO follicle development *in vivo* and *in vitro*.

Comparing how the age of ovaries affected subsequent follicle development revealed differences when comparing P2 and P6 ROs transplanted for 14 days. Interestingly, the follicle populations present in P2-ROt + 14d and P6-ROt + 14d resembled the different follicle populations originally in P2 and P6 ovaries (P2-O and P6-O) respectively. This could be indicating a delay while the oocytes and somatic cells reform follicles and restore communication, as previously hypothesised (Park *et al.* 2006) or accelerated follicle development in P6 ROs due to advanced cellular development in P6 ovaries. The difference in follicle development between P2 and P6 ROs after 21 days of transplantation is not as pronounced as 14 days, but there is a trend for further follicle development in P6 ROs, as evidenced by the increased number of developing follicles. Therefore, our data are also consistent with Eppig’s work indicating the stage of oocyte development is retained (Eppig *et al.* 2002) when follicles are developed in the same condition. Additionally, the ovarian dissociation involved in RO generation unsurprisingly results in putative primordial oocytes without a defined layer of pre-GCs throughout the RO. Fragmentation of ovarian tissue results in primordial follicle activation via the Hippo signalling pathway (Kawamura *et al.* 2013). However, the presence of putative primordial follicles in the ROs after 14 days of culture or transplantation indicates that not all follicles are activated by the dissociation procedure and thus a primordial pool is available for subsequent follicle development.

When comparing transplanted and cultured ovaries and ROs, follicle development *in vivo* differed markedly to those developed in culture. In the cultured ROs, unlike the transplanted ROs, follicles developed up to the preantral stage, furthermore, an age-related difference in follicle profile (i.e., P2 vs P6) was not observed. The acceleration of follicle development in culture has previously been observed in static ovary culture. However, it is unclear whether the acceleration in follicle development is due to growth-promoting factors in culture compared to *in vivo* conditions (Fortune *et al.* 2000), whether the ‘brakes’ controlling follicle development are absent or non-functional in culture (Roness *et al.* 2013) or a combination of the two. The acceleration in follicle development was similar between the ROs and whole ovaries cultured for 14 days, unlike Park *et al*. (2006) who noted less development in the ROs after just 4 days of culture. This indicates that ROs cultured for 14 days develop follicles to the preantral stage despite starting culture as a mixture of oocytes and somatic cells, rather than the organised structure as is the case for whole ovaries. Therefore, although the dissociation of ovaries for RO generation likely alters the Hippo signalling pathway (Kawamura *et al.* 2013), ECM and other cell–cell interactions, this does not override the environment the tissue is cultured in when assessing follicle development.

The most striking difference in follicle development when comparing cultured ROs and ovaries to their *in vivo* counterparts is the reduced number of TCs surrounding follicles. The later stages of follicle development are dependent on TC association with the follicle (Spears *et al.* 1994). However, a number of defects in follicle structure have been observed in follicles from organ culture, including a lack of a complete FBL, a less defined theca layer (Eppig & O’Brien 1996), along with fewer proliferating GCs and smaller primary and secondary oocytes (Wang *et al.* 2013); these findings are mirrored in our results. Smaller oocytes in cultured ovaries and ROs, as we observed, may also be indicative of slower oocyte growth (Griffin *et al.* 2006), which may affect the numbers of GC and TC associated and potentially FBL formation. In addition, the lack of FBL may also affect GC proliferation and differentiation (Irving-Rodgers & Rodgers 2006). Slower proliferating GCs in follicles from cultured ROs and ovaries may also contribute to fewer TCs observed, as they secrete theca recruitment factors such KITL and EGF (reviewed by Young & McNeilly 2010). This triumvirate of poor FBL, fewer GCs and fewer TCs may result in a negative feedback loop, leading to poorer follicle quality *in vitro*.

As both transplanted ROs and *in vivo* Control ovaries have the same cellular components as cultured ROs and ovaries respectively, the differences in follicle development must be attributed to the environment they are grown in. Since the main environmental difference between transplanted ROs and cultured ROs is exposure to vasculature, a variety of factors could be added to optimise follicle development in culture. For example, insulin has been identified as having a role in TC proliferation and differentiation (reviewed in (Young & McNeilly 2010); however, insulin was included in our culture system (ITS) and did not rescue theca cell development. However, other factors may affect TCs development in culture. Follicle co-culture with mesenchymal stem cells (MSCs) has been shown to improve preantral follicle development (Xia *et al.* 2015). The MSCs are thought to be promoting follicle development by acting as a ‘drugstore’ for the follicle and secreting factors such as activin A, transforming growth factor, vascular endothelial growth factor and fibroblast growth factor (Xia *et al.* 2015), which are also known to promote theca development (Young & McNeilly 2010). Therefore, the lack of TC, GC and FBL development *in vitro* suggests that additional factors, be they physical or molecular, are needed to improve them in culture.

The results presented here highlight several important aspects of *in vitro* follicle development that are particularly relevant given the increased interest in gamete development from ROs (Hikabe *et al.* 2016). Although cultured ROs contained the same cells as their *in vivo* counterparts prior to culture, the morphological differences, such as fewer TCs and acceleration in follicle development highlights the need for further understanding of follicle development in culture. The difference in follicle development between transplanted P2 and P6 ROs also demonstrates that the oocyte contains the intrinsic developmental programme *in vivo* (Eppig *et al.* 2002). However, this intrinsic programming and difference in P2 and P6 ovarian physiology is overridden in cultured ROs. Finally, the mechanism behind the reformation of follicles in ROs is unknown: are the somatic cells surrounding the oocyte dedifferentiated or transdifferentiated into the cells that belong in that niche (reviewed by Merrell & Stanger 2016) or do they reassemble based on their differentiated phenotype (Campbell *et al.* 2013, Teng *et al.* 2015).

Despite these alterations in follicle development in cultured ROs, the RO technique may prove to be useful in understanding follicle development and perhaps even provide an alternative method of ovarian tissue culture for patients with functionally suboptimal somatic cells. Patients who may benefit include those with premature ovarian insufficiency (POI; 1% of women below 40 years of age, (Coulam *et al.* 1986) who still have follicles present within their ovaries or whose ovaries are diseased, such as in granulosa cell tumours (GCTs) (Rantala 1988, Unkila-Kallio *et al.* 2000). In these instances, the carefully regulated process of follicle development does not, or cannot, proceed *in vivo*. By replacing these patients’ somatic cells with an alternative source, follicle development using their own germ cells may proceed within an RO.

Our results highlight the flexibility of the ovary; even after dissociation and reaggregation, follicles can be reformed, with cellular compartments within follicles and FBL definition in transplanted ROs comparable to that of *in vivo* ovaries. However, this resilience and follicle reformation is highly dependent on the environment, as evidenced by the poorer FBL definition, and changes in TC and GC number in cultured ROs. Furthermore, follicle development *in vitro* also appears to act independently of the differences in ovarian physiology, in terms of stage of oocyte nest breakdown and primordial follicle formation within the source ovaries. By understanding the mechanisms by which follicles reform within the RO, *in vitro* follicle development can be improved and enhance ovarian tissue culture methods.

## Declaration of interest

Suzannah Williams is a member of the Editorial Board of Reproduction. The other authors declare no conflict of interest that could be perceived as prejudicing the impartiality of the research reported.

## Funding

This work was supported by a MRC Centenary Early Career Award (G0900058/1) to S A W, a Clarendon Fund Scholarship and Nuffield Department of Obstetrics and Gynaecology funding to B K M L, and a Leverhulme Postgraduate Bursary and EPA Cephalosporin Scholarship from Linacre College, University of Oxford, to S S.

## Author contribution statement

B K M L and S A W designed the work, with input from S S. Experiments and analysis was performed by B K M L. All surgeries were performed by S S. The manuscript was written by B K M L, S S and S A W. All authors have read and approved the final manuscript.
